# Phylogenetic inference of inter-population transmission rates for infectious diseases

**DOI:** 10.1093/bib/bbae312

**Published:** 2024-06-26

**Authors:** Skylar A Gay, Gregory Ellison, Jianing Xu, Jialin Yang, Yiliang Wei, Shaoyuan Wu, Lili Yu, Christopher C Whalen, Jonathan Arnold, Liang Liu

**Affiliations:** Institute of Bioinformatics, University of Georgia, 120 Green Street, Athens, GA 30602, United States; Department of Statistics, University of Georgia, 310 Herty Drive, Athens, GA 30602, United States; Department of Statistics, University of Georgia, 310 Herty Drive, Athens, GA 30602, United States; Department of Statistics, University of Georgia, 310 Herty Drive, Athens, GA 30602, United States; Jiangsu Key Laboratory of Phylogenomics and Comparative Genomics, Jiangsu International Joint Center of Genomics, School of Life Sciences, Jiangsu Normal University, 101 Shanghai Road, Xuzhou, Jiangsu 221116, China; Jiangsu Key Laboratory of Phylogenomics and Comparative Genomics, Jiangsu International Joint Center of Genomics, School of Life Sciences, Jiangsu Normal University, 101 Shanghai Road, Xuzhou, Jiangsu 221116, China; Department of Biostatistics, Epidemiology and Environmental Health Sciences, College of Public Health, Georgia Southern University, 1332 Southern Drive, Statesboro, GA 30677, United States; Global Health Institute, Department of Epidemiology and Biostatistics, College of Public Health, University of Georgia, 100 Foster Road, Athens, GA 30602, United States; Institute of Bioinformatics, University of Georgia, 120 Green Street, Athens, GA 30602, United States; Department of Genetics, University of Georgia, 120 West Green Street, Athens, GA 30602, United States; Institute of Bioinformatics, University of Georgia, 120 Green Street, Athens, GA 30602, United States; Department of Statistics, University of Georgia, 310 Herty Drive, Athens, GA 30602, United States

**Keywords:** phylogenetic tree, SIR model, infectious disease, COVID-19, transmission rate

## Abstract

Estimating transmission rates is a challenging yet essential aspect of comprehending and controlling the spread of infectious diseases. Various methods exist for estimating transmission rates, each with distinct assumptions, data needs, and constraints. This study introduces a novel phylogenetic approach called transRate, which integrates genetic information with traditional epidemiological approaches to estimate inter-population transmission rates. The phylogenetic method is statistically consistent as the sample size (i.e. the number of pathogen genomes) approaches infinity under the multi-population susceptible-infected-recovered model. Simulation analyses indicate that transRate can accurately estimate the transmission rate with a sample size of 200 ~ 400 pathogen genomes. Using transRate, we analyzed 40,028 high-quality sequences of SARS-CoV-2 in human hosts during the early pandemic. Our analysis uncovered significant transmission between populations even before widespread travel restrictions were implemented. The development of transRate provides valuable insights for scientists and public health officials to enhance their understanding of the pandemic’s progression and aiding in preparedness for future viral outbreaks. As public databases for genomic sequences continue to expand, transRate is increasingly vital for tracking and mitigating the spread of infectious diseases.

## Introduction

Assessing transmission rates is essential for understanding the dynamics of infectious diseases [1, 2]. By gaining insights into transmission rates, public health officials can create strategies to mitigate the impact of a disease outbreak and be prepared for potential future outbreaks [3, 4]. Various techniques and approaches have been devised to estimate transmission rates using epidemiological and genetic data in infectious diseases [5–9]. Conventional approaches, such as the computation of the basic reproduction number ${\boldsymbol{R}}_{\mathbf{0}}$, represent some of the most straightforward techniques for assessing transmission rates. Estimation of ${\boldsymbol{R}}_{\mathbf{0}}$ involves examining the growth rate of the epidemic curve under the susceptible-infectious-recovered (SIR) and susceptible-exposed-infectious-recovered (SEIR) models with the assumptions of a consistent transmission rate and a homogeneous population [10]. Several methods have been developed to account for temporal variations in estimating transmission rates [11–13]. The performance of these methods relies on the assumption that symptom onset accurately reflects the date of infection, which might not hold true for cases of asymptomatic transmission [14].

Analyzing epidemiological data offers valuable insights into the transmission dynamics of a disease by fitting models to observed data and estimating relevant parameters [15–18]. Since transmission rates can exhibit spatial variability [19], analyzing the disease spread across different regions is instrumental in understanding the spatial distribution of transmission rates [20, 21]. Furthermore, advancements in genomic sequencing technologies have enabled researchers to trace the dissemination of pathogens at a molecular level [22]. Phylogenetic trees constructed from genetic data have emerged as fundamental tools to elucidate the relatedness of different pathogen strains [23]. By scrutinizing the branching patterns of the tree, researchers can deduce transmission dynamics, including the direction and frequency of transmission events [7].

**Figure 1:**
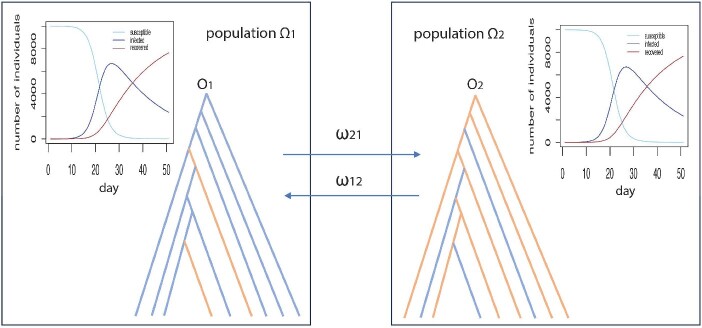
Transmission events generated from the two-population SIR model; the numbers of susceptible we consider the transmission events that occur within and between two populations ${\Omega}_1$ (left panel) and ${\Omega}_2$ (right panel); the numbers of susceptible, infected, and recovered individuals at time $t$ (day) were obtained by solving the differential equations for the two-population SIR model; moreover, the model assumes that transmissions occur between two populations at a constant rate ${\omega}_{12}$ for transmissions from the population ${\Omega}_1$ to the population ${\Omega}_2$ and ${\omega}_{21}$ for transmissions from the population ${\Omega}_2$ to the population ${\Omega}_1$, and every infected individual in the population ${\Omega}_1$ can be traced back to an infector (i.e. ancestor) in the population ${\Omega}_1$; the ancestral history of all transmissions in the population ${\Omega}_1$ form a phylogenetic tree (left panel) with a root ${O}_1$ in which most lineages are the transmissions within the population ${\Omega}_1$ and two lineages are the inter-population transmissions from the population ${\Omega}_2$ to the population ${\Omega}_1$; similarly, a phylogenetic tree with a root ${O}_2$ (right panel) can be generated for the transmissions in the population ${\Omega}_2$ where most lineages are the transmissions within the population ${\Omega}_2$ and three lineages are the inter-population transmissions from the population ${\Omega}_1$ to the population ${\Omega}_2$.

Traditional methods for estimating transmission rates have predominantly centered on tracking the spread of infectious diseases within a single population. This paper introduces a novel approach by presenting a multi-population SIR model for analyzing transmission rates within and between populations (see Fig. 1). Leveraging this multi-population SIR model, we have developed a phylogenetic method to accurately estimate inter-population transmission rates. Our aim is to furnish a holistic comprehension of transmission dynamics across multiple populations for infectious diseases.

## Materials and Methods

### Modeling transmissions for multiple populations

The multi-population SIR model, an extension of the traditional SIR model [24], serves to simulate disease transmissions across multiple populations (S1 in Supplementary data). This model can be readily expanded into more intricate epidemiological models. For instance, it can adopt the SEIR model framework, which, compared to the SIR model, provides a more accurate representation of infectious diseases with discernible incubation periods [25]. However, since infection and recovery rates are two pivotal parameters in the formula of estimating inter-population transmission rates, the multi-population SIR model is deemed sufficient for accurately estimating these rates. In the multi-population SIR model, transmission events occur within and between $K$ populations ${\Omega}_1,\dots, {\Omega}_K$ of size ${N}_1,\dots, {N}_K$ during a time interval $\left[0,d\right]$. Let ${S}_{t,k},{I}_{t,k},{R}_{t,k}$ be the number of susceptible, infectious, and recovered individuals at time $t$ for the population $k=1,\dots, K$. The variables ${S}_{t,k},{I}_{t,k},{R}_{t,k}$ in each population satisfy the differential equations of the SIR model for a single population (S1 in Supplementary data), i.e.


(1) \begin{align*} \left\{\begin{array}{@{}l}\frac{d{S}_{t,k}}{dt}=-{\beta}_k{I}_{t,k}{S}_{t,k}\kern0.50em \ \kern0.50em \ \\[3pt] {}\frac{d{I}_{t,k}}{dt}={\beta}_k{I}_{t,k}{S}_{t,k}-{\gamma}_k{I}_{t,k}\\[3pt] {}\frac{d{R}_{t,k}}{dt}={\gamma}_k{I}_{t,k}\kern0.50em \ \kern0.50em \ \kern0.50em \ \kern0.50em \ \kern0.50em \ \ \end{array}\right.. \end{align*}

Moreover, transmissions occur between two populations $i$ and $j$ at a constant rate ${\omega}_{ij}$ for transmissions from population ${\Omega}_j$ to population ${\Omega}_i$ and ${\omega}_{ji}$ for transmissions from population ${\Omega}_i$ to population ${\Omega}_j$ (Fig. 1). The inter-population transmission rate ${\omega}_{ij}$ represents the probability of an individual from population ${\Omega}_j$ traveling to population ${\Omega}_i$ and contracting the infection in ${\Omega}_i$, i.e.


(2) \begin{align*} {\omega}_{ij}= wv, \end{align*}

where $w$ is the probability that an individual in population ${\Omega}_j$ travels to population ${\Omega}_i$, and $v$ is the probability that an individual who has traveled to population ${\Omega}_i$ gets infected in ${\Omega}_i$. If individuals in population ${\Omega}_j$ independently have the same probability $w$ of traveling to population ${\Omega}_i$, then the number ${y}_{t,j}$ of individuals in population ${\Omega}_j$ who travel to population ${\Omega}_i$ at time $t$ follows the binomial distribution, i.e.


(3) \begin{align*} {y}_{t,j}\sim Binomial\left({N}_j,w\right). \end{align*}

Given ${y}_{t,j}$, the number ${x}_{t,j}$ of individuals in population ${\Omega}_j$ who have traveled to and gotten infected in population ${\Omega}_i$ at time $t$ follows the binomial distribution, i.e.


(4) \begin{align*} {x}_{t,j}\mid{y}_{t,j}\sim Binomial\left({y}_{t,j}{I}_{t,i},v\right). \end{align*}

The transmission events occurring in population ${\Omega}_i$ involve not only the infected individuals in the population ${\Omega}_i$ but also the ${x}_{t,j}$ individuals from population ${\Omega}_j$ who travel to and get infected in ${\Omega}_i$. Every newly infected individual ${\mathcal{I}}_{t,i}$ in ${\Omega}_i$ at time $t$, including the ${x}_{t,j}$ individuals who have traveled from ${\Omega}_j$ to ${\Omega}_i$, can be traced back to an infectious individual ${\mathcal{A}}_{t-1,i}$ in ${\Omega}_i$ at time $t-1$. This transmission event is indicated by a mapping $\tau$ defined as follows:


(5) \begin{align*} \tau :{\mathcal{I}}_{t,i}\mapsto{\mathcal{A}}_{t-1,i} \end{align*}

Since the number ${x}_{t,j}$ is negligible compared to the number ${I}_{t-1,i}$ of infectious individuals in the population ${\Omega}_i$ at time $t-1$, we only consider the ${I}_{t-1,i}$ infectious individuals when we look backward to find the infectious individual ${\mathcal{A}}_{t-1,i}$ at time $t-1$ who is the ancestor of a newly infected individual ${\mathcal{I}}_{t,i}$ at time $t$. Furthermore, it is assumed that the ${I}_{t-1,i}$ infectious individuals at time $t-1$ are equally likely to be the ancestor ${\mathcal{A}}_{t-1,i}$ of a newly infected individual ${\mathcal{I}}_{t,i}$, i.e. for $a=1,\dots, {I}_{t-1,i}$, where $a$ represents one of the ${I}_{t-1,i}$ infectious individuals in population $i$ at time $t-1$,


(6) \begin{align*} P\left({\mathcal{A}}_{t-1,i}=a\right)=\frac{1}{I_{t-1,i}} . \end{align*}

Moreover, the ${x}_{t,j}$ individuals from population ${\Omega}_j$ are infected by the ${I}_{t,i}$ infectious individuals of population ${\Omega}_i$ at time $t$. We assume that the ${I}_{t,i}$ infectious individuals at time $t$ are equally likely to be the ancestor ${\mathcal{A}}_{t,i}$ of one of the ${x}_{t,j}$ individuals from population ${\Omega}_j$, i.e. for $b=1,\dots, {I}_{t,i}$, where $b$ represents one of the ${I}_{t,i}$ infectious individuals


(7) \begin{align*} P\left({\mathcal{A}}_{t,i}=b\right)=\frac{1}{I_{t,i}}. \end{align*}

All transmissions within an arbitrary time interval [0, ${d}_i$] for ${d}_i>2$ and ${d}_i\in \mathbb{N}$ in population ${\Omega}_i$ form a tree-like structure, which is the transmission tree ${T}_i$ for population ${\Omega}_i$ (Fig. 1). We assume that the roots ${O}_1,\dots, {O}_K$ of $K$ transmission trees ${T}_1,\dots, {T}_K$ share a common ancestor, denoted by ${O}^{\ast }$, i.e. the root of a super tree ${T}^{\ast }$ in which the $K$ transmission trees ${T}_1,\dots, {T}_K$ are the subtrees of ${T}^{\ast }$.

It follows from Equations 3 and 4 that the expected number $E\left({x}_{t,j}\right)$ of individuals who travel to and get infected in the population ${\Omega}_i$ is given by $E\left({x}_{t,j}\right)=E\left(E\left({x}_{t,j}|{y}_{t,j}\right)\right)={N}_j{I}_{t,i} wv={N}_j{I}_{t,i}{\omega}_{ij}$, where ${I}_{t,i}$ is the number of infectious individuals in the population ${\Omega}_i$ at time $t$. The expectation of the total number ${\sum}_t{x}_{t,j}$ of individuals from the population ${\Omega}_j$ who get infected in the population ${\Omega}_i$ is equal to $E\left({\sum}_t{x}_{t,j}\right)={N}_j{\omega}_{ij}{\sum}_t{I}_{t,i}$, indicating that the transmission rate ${\omega}_{ij}$ can be estimated by the ratio of ${\sum}_t{x}_{t,j}$ and ${N}_j{\sum}_t{I}_{t,i}$, i.e.


(8) \begin{align*} \hat{\omega_{ij}}=\frac{\sum_t{x}_{t,j}}{N_j{\sum}_t{I}_{t,i}}. \end{align*}

The numerator ${\sum}_t{x}_{t,j}$ is the total number of individuals from the population ${\Omega}_j$ who travel to and get infected in the population ${\Omega}_i$. The denominator ${N}_j{\sum}_t{I}_{t,i}$ can be calculated by ${N}_j{\sum}_t{I}_{t,i}={N}_j{\sum}_{m=1}^{I_i}({t}_{m,i}^R-{t}_{m,i}^I)$ where ${t}_{m,i}^R$ and ${t}_{m,i}^I$ are the recovery and infection time of the infected individual $m$ in the population ${\Omega}_i$, and ${I}_i$ is the total number of infected individuals in the population ${\Omega}_i$ by time ${d}_i$. Thus, the estimate $\hat{\omega_{ij}}$ can be calculated by


(9) \begin{align*} \hat{\omega_{ij}}=\frac{\sum_t{x}_{t,j}}{N_j{\sum}_{m=1}^{I_i}\left({t}_{m,i}^R-{t}_{m,i}^I\right)}. \end{align*}

The estimate $\hat{\omega_{ij}}$ is unbiased and statistically consistent in estimating the inter-population transmission rate ${\omega}_{ij}$ (S2 and S3 in Supplementary data).

In real data analysis, however, we can only obtain a sample of infected individuals in populations ${\Omega}_1,\dots, {\Omega}_K$. We assume that the infected individuals in the samples ${\mathcal{S}}_1$,…,${\mathcal{S}}_K$ are randomly selected from populations ${\Omega}_1,\dots, {\Omega}_K$. Let ${n}_i$ be the sample size of ${\mathcal{S}}_i$, and ${\tilde{x}}_{t,j}$$\left(j\ne i\right)$ denotes the number of individuals in the sample ${\mathcal{S}}_j$ who travel to and get infected population ${\Omega}_i$ at time $t$. Let $\tilde{I_i}$ for $i=1,\dots, K$ be the number of individuals in the samples ${\mathcal{S}}_i$ who get infected in population ${\Omega}_i$. Let ${I}_i$ be the total number of infected individuals by the time ${d}_i$ in population ${\Omega}_i$. The inter-population transmission rate ${\omega}_{ij}$ can be estimated by the samples ${\mathcal{S}}_i$ and ${\mathcal{S}}_j$, i.e.


(10) \begin{align*} \tilde{\omega_{ij}}=\frac{\frac{I_j}{\tilde{I_j}}{\sum}_t{\tilde{x}}_{t,j}}{N_j\left(\frac{I_i}{\tilde{I_i}}{\sum}_{m=1}^{\tilde{I_i}}\left({t}_{m,i}^R-{t}_{m,i}^I\right)\right)}. \end{align*}

The estimate $\tilde{\omega_{ij}}$ converges to $\hat{\omega_{ij}}$, i.e. $\tilde{\omega_{ij}}\to \hat{\omega_{ij}}$, as the sample sizes ${n}_i$ and ${n}_j$ approach to the total numbers ${N}_i$ and ${N}_j$ of the infected individuals in the populations ${\Omega}_i$ and ${\Omega}_j.$ We can show that $\tilde{\omega_{ij}}$ is an asymptotically unbiased estimator of ${\omega}_{ij}$ and is statistically consistent in estimating the parameter ${\omega}_{ij}$ as the sample sizes ${n}_1$ and ${n}_2$ increase to infinity (S4 in Supplementary data).

We have developed a phylogenetic approach (transRate) to estimate the inter-population transmission rate ${\omega}_{ij}$ using the pathogen genomes labeled with their population origins ${\Omega}_1,\dots, {\Omega}_K$. The phylogenetic method for transmission rate estimation consists of four steps: (i) building a phylogenetic tree based on the pathogen genomes. (ii) Identifying clades in the tree that have at least a certain percentage $\left(\alpha \right)$ of taxa with the same population origin. By default, $\alpha$ is set at 60%. Nonetheless, users have the flexibility to adjust $\alpha$ such that the count of strongly supported clades aligns with the number of populations. (iii) Labeling each identified clade with the population origin of the majority sequences in that clade. Sequences labeled with a distinct population origin are inferred as instances of inter-population transmission. (iv) Estimating the inter-population transmission rate ${\omega}_{ij}$ based on the identified clades. The variability inherent in the estimation of phylogenetic trees, including the formation of clades, could skew the accuracy of transmission rate estimation. However, given the constancy of the transmission rate over time, the percentage of transmission events remains the same throughout any given time frame. This indicates that, despite the inherent uncertainty in the phylogenetic tree and the identification of clades, the estimation of the transmission rate retains a certain degree of reliability.

### Simulation

#### Estimation of transmission rates from phylogenetic trees

Given the transmission tree of two populations, we evaluated the performance of transRate for estimating the inter-population transmission rate. The transmission tree was generated from the two-population SIR model during a time interval $\left[0,d\right]$. The population size was designated as ${N}_1={N}_2=10,000$ and ${N}_1={N}_2=1,000,000$. For the population size $10,000$, we set the infection rate $\beta =5\times{10}^{-5}$ and the recovery rate $\gamma =0.05$. For the population size $1,000,000$, we set $\beta =5\times{10}^{-7}$ and $\gamma =0.05$. The values of the infection and recovery rates were based on the estimates obtained from the early stages of the COVID-19 pandemic. An infection rate ($\beta$) of $5\times{10}^{-5}$ for a population of 10,000 suggest rapid infection with the entire population being infected within 30 days (Fig. 1). The recovery rate ($\gamma$) of 0.05 indicates a fast recovery with an average recovery time of 20 days. The number of susceptible (${S}_t)$, infected $\left({I}_t\right)$, and recovered $\left({R}_t\right)$ at time $t$ was obtained by solving the differential equations of the two-population SIR model using an R package deSolve [26]. The number ${y}_{t,2}$of individuals who traveled from the population ${\Omega}_2$ to the population ${\Omega}_1$ at time $t\in \left[0,50\right]$ was simulated from the binomial distribution with mean = ${N}_2w$, where $w=0.0001$ for the population size $\mathrm{10,000}$ and $w=0.000001$ for the population size $1000000$. The parameter $w$ is the probability that an individual in the population ${\Omega}_2$ travels to the population ${\Omega}_1$, i.e. the average number of travelers from the population ${\Omega}_2$ to the population ${\Omega}_1$ of size 10,000 is $10000\times 0.0001=10$ individuals per day. Given ${y}_{t,2}$, the number ${x}_{t,2}$ of individuals who traveled to and were infected in the population ${\Omega}_1$ at time $t$ was simulated from the binomial distribution with the infection rate $v=0.002,0.004,0.006,0.008$. The inter-population transmission rate ${\omega}_{12}$ from the population ${\Omega}_2$ to the population ${\Omega}_1$ is equal to the product of two probabilities $w$ and $v$, i.e. ${\omega}_{12}= wv=2\times{10}^{-7},4\times{10}^{-7},6\times{10}^{-7},8\times{10}^{-7}$ for the population size 10000 and ${\omega}_{12}= wv=2\times{10}^{-9},4\times{10}^{-9},6\times{10}^{-9},8\times{10}^{-9}$ for the population size 1000000, respectively. Similarly, the transmissions from the population ${\Omega}_1$ to the population ${\Omega}_2$ were simulated with the transmission rate ${\omega}_{21}$. Two inter-population transmission rates were assumed to be equal, i.e. ${\omega}_{12}={\omega}_{21}$.

To understand the transmission dynamics of other pathogens with varying transmission modes, we simulated data for a population of 10,000 with slower infection and recovery rates, i.e. $\beta =5\times{10}^{-6}$ and $\gamma =0.005$. Compared with fast infection, the reduced infection rate of $\beta =5\times{10}^{-6}$ would require 300 days for the entire population of 10,000 to become infected, while the slower recovery rate of $\gamma =0.005$ indicated an average recovery period of 200 days (S5 in Supplementary data). The number of susceptible (${S}_t)$, infected $\left({I}_t\right)$, and recovered $\left({R}_t\right)$ at time $t$ was obtained by solving the differential equations of the two-population SIR model. The number of individuals who traveled from the population ${\Omega}_2$ to the population ${\Omega}_1$ at time $t\in \left[0,500\right]$ was simulated from the binomial distribution $\left(n=10000,p=0.0001\right)$. The inter-population transmission rates ${\omega}_{12}$ and ${\omega}_{21}$ were designated as ${\omega}_{12}={\omega}_{21}=2\times{10}^{-8},4\times{10}^{-8},6\times{10}^{-8},8\times{10}^{-8}$.

A phylogenetic tree ${T}_1$ was subsequently constructed from the simulated transmissions in the population ${\Omega}_1$. Let ${x}_i$ be the number of new infections on Day $i$. Let ${y}_{i-1}$be the number of infections on Day ($i-1$). Note that ${x}_i$ and ${y}_{i-1}$ included inter-population infections, and the ${x}_i$ new infections on day $i$ were infected by the ${y}_{i-1}$ infectious individuals on Day $\left(i-1\right)$. Since the infectious individuals on day $\left(i-1\right)$ were equally likely to infect the susceptible individuals on Day $i$, the ancestor of each new infection on day $i$ was found by randomly sampling an infectious individual on day $\left(i-1\right)$. The ancestors formed the ancestral history (i.e. the phylogenetic tree ${T}_1$) of the transmissions in the population ${\Omega}_1$ generated from the two-population SIR model. Similarly, a phylogenetic tree ${T}_2$ was constructed from the transmissions in the population ${\Omega}_2$. Two trees were combined into a super tree $T$. This super tree $T$ was the input to estimate the transmission rates ${\omega}_{12}$ and ${\omega}_{21}$ using Equation 9. Moreover, we randomly selected $n=100,200,300,400,500$ infected individuals (taxa) from each population in the super tree $T$. The phylogenetic tree of the sampled infections was utilized to estimate the inter-population transmission rates ${\omega}_{12}$ and ${\omega}_{21}$ using Equation 10. Each simulation was repeated 100 times and we calculated the mean squared error (MSE) and coefficient variation (CV) of the estimates of the transmission rate. Since two transmission rates are equal to each other, we only present the MSE and CV of the transmission rate ${\omega}_{12}$, i.e. $MSE=\frac{1}{100}{\sum}_{i=1}^{100}{({\hat{\omega_{12}}}^i-{\omega}_{12})}^2$ and $CV=\frac{sd\left(\hat{\omega_{12}}\right)}{mean\left(\hat{\omega_{12}}\right)}$, where $sd\left(\hat{\omega_{12}}\right)$ is the standard deviation of $\hat{\omega_{12}}$.

#### Estimation of transmission rates from molecular sequences

In the preceding simulation, the phylogenetic tree was derived from the transmissions generated by the two-population SIR model and was assumed to be a known input for estimating the inter-population transmission rate ${\omega}_{12}$. However, in practice, the phylogenetic tree is typically inferred from the alignments of pathogen genomes. Therefore, it becomes crucial to account for the uncertainty associated with the estimated phylogenetic tree when estimating the transmission rate ${\omega}_{12}$. Once the phylogenetic tree was constructed based on the transmissions generated from the two-population SIR model, we proceeded to simulate deoxyribonucleic acid (DNA) sequences of 20,000 base pairs using the phylogenetic program Seq-Gen [27] with the mutation rate $\mu =0.01,0.001,0.0001$. These sequences were utilized to reconstruct the maximum likelihood (ML) tree using FastTree [28]. The estimated phylogenetic tree served as the input for inferring the transmission rate ${\omega}_{12}$. Each simulation was repeated 100 times and we calculated the MSE and co-efficient variation (CV) of the estimates of the transmission rate ${\omega}_{12}$.

#### Estimation of transmission rates for multiple populations

In this simulation, transmissions were generated from the multi-population SIR model for five populations ${\Omega}_1,{\Omega}_2,{\Omega}_3,{\Omega}_4,{\Omega}_5$. The populations were characterized by two different sizes, one with 10,000 individuals and another with 1,000,000 individuals. For the smaller population (10,000 individuals), transmission rates (${\omega}_{ij}$ for $i,j=1,\dots, 5$) were set at values of $2\times{10}^{-7},\kern0.5em 4\times{10}^{-7},6\times{10}^{-7}$ and $8\times{10}^{-7}$, while for the larger population (1,000,000 individuals), transmission rates were configured at $2\times{10}^{-9},\kern0.5em 4\times{10}^{-9},6\times{10}^{-9}$, and $8\times{10}^{-9}$. A sample of infected individuals (i.e. ${n}_1={n}_2={n}_3={n}_4={n}_5=100,200,300$) was randomly selected from each of the five populations. Due to the limitation of the phylogenetic tree reconstruction method, we did not sample more than 300 individuals. The selected individuals were labeled with their population origins. Subsequently, a phylogenetic tree was constructed from the transmissions among the five samples of infected individuals. This phylogenetic tree featured five major clades, each corresponding to the sample selected from one of the five populations. DNA sequences of 20,000 base pairs were simulated from the phylogenetic tree using Seq-Gen. These simulated sequences were employed as input data to estimate ML trees using the FastTree algorithm. The ML tree, in turn, was utilized to infer the transmission rates (${\omega}_{ij}$). Finally, we evaluated the performance of transRate by calculating the MSE and CV of the estimates of the transmission rates (${\omega}_{ij}$). The MSE and CV of the transmission rate estimates served as a measure of how well the phylogenetic approach performed in estimating transmission rates in the simulation.

### Data analysis of SARS-CoV-2 genomes in the early pandemic

The proposed phylogenetic method transRate was applied to a genomic dataset consisting of 40,028 sequences of SARS-CoV-2 in human hosts during the early SARS-CoV-2 pandemic [29]. This dataset was formed from 41,910 coronavirus genomes retrieved from NCBI GenBank nucleotide database on 26 August 2021, with all sequences collected from regions across Asia, Europe, Oceania, Africa, and the Americas between 31 December 2019, and 1 April 2020. The dataset was filtered to remove sequences with missingness, containing frame shift, and incomplete genomes. The filtration returned a dataset of 40,028 SARS-CoV-2 genomes isolated from human hosts. The genomes were annotated with the geographical locations corresponding to where the samples were collected. The sequence datasets for each of the 11 genes in SARS-CoV-2 and the full-genome sequence dataset were constructed and aligned, whereafter ML and bootstrap phylogenetic trees were built from the 11 gene alignments using RAxML [30] with the GTRCAT model. The species tree was estimated from ML and bootstrap gene trees using a coalescent method NJst [31]. The geographical distribution of the clades in the species tree are as follows: 11 clades geographically centered in the Americas, 5 clades geographically centered in Asia, 18 clades geographically centered in Europe, and 1 clade geographically centered in Oceania (S7 in Supplementary data). A number of these clades did not contain any geographical outliers. A group of datasets were formed for the following populations: “Africa” (includes any clades that were centered in countries within the continent of Africa), “Americas” (includes any clades that were centered in countries or localities that were in North, Central, and South America), “Asia” (includes any clades that were centered in countries located in Asia), “Europe” (includes any clades that were centered in countries located in Europe), and “Oceania” (includes any clades that were centered in localities in Oceania). The transmission rate estimation calculation was applied to the above populations. The recovery time is equal to 14 days as initially issued by the World Health Organization in the early pandemic. Population data was collected based on the World Health Organization and United Nations published data (S8 in Supplementary data).

A transmission analysis airplane plot was constructed using the previously described dataset of 40,028 whole genome sequences of SARS-CoV-2 in human hosts. These samples underwent the process of forming clades based on bootstrap support, location, and number of individuals per clade, as outlined above. The inferred transmission events were illustrated in an airplane plot with the larger black dots representing the location of which the majority of clade members originated. The smaller black dots are geographical outliers (samples not from within the location of the majority) in the clade and the arcs connecting the clade center to the geographical outliers represent an inferred transmission event. The color of the arcs indicates the time point at which the inferred transmission event occurred. This plot was created using the maps (Becker et al, 2022) and geosphere (Hijmans et al, 2022) packages in R.

## Results

### Simulation

 

### Estimation of transmission rates from phylogenetic trees

**Figure 2:**
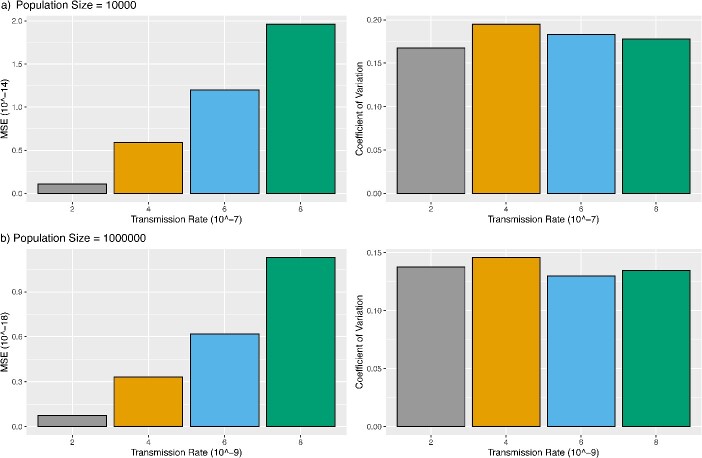
Estimation of the transmission rate from the phylogenetic tree of all infected individuals; the phylogenetic tree of all infected individuals was generated from the two-population SIR model; (a) for population size = 10,000, transmission events were simulated with the transmission rate $\omega =2\times{10}^{-7},4\times{10}^{-7},6\times{10}^{-7},8\times{10}^{-7}$, and (b) for population size = 1,000,000, transmission events were simulated with the transmission rate $\omega =2\times{10}^{-9},4\times{10}^{-9},6\times{10}^{-9},8\times{10}^{-9}$; the phylogenetic tree was then utilized to estimate the transmission rate $\omega$, and the simulation was repeated 100 times; the MSE and CV of the transmission rate estimates were calculated.

**Figure 3:**
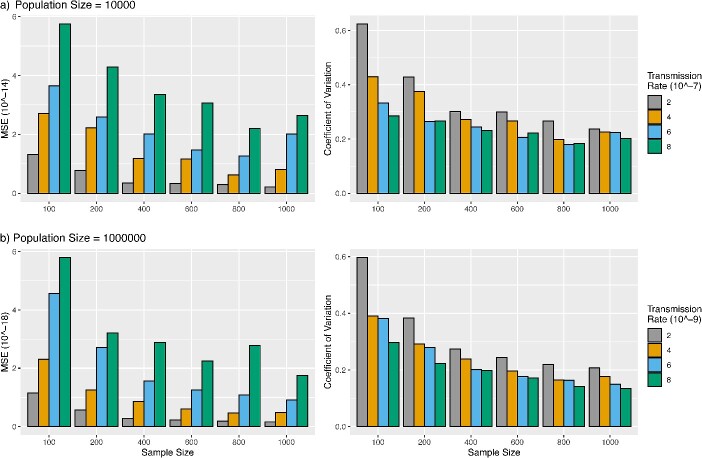
Estimation of the transmission rate from the phylogenetic tree of a sample of infected individuals; the phylogenetic tree of a sample of infected individuals (sample size = 100, 200, 400, 600, 800, 1000) was generated from the two-population SIR model; (a) for population size = 10,000, transmission events were simulated with the transmission rate $\omega =2\times{10}^{-7},4\times{10}^{-7},6\times{10}^{-7},8\times{10}^{-7}$, and (b) for population size = 1,000,000, transmission events were simulated with the transmission rate $\omega =2\times{10}^{-9},4\times{10}^{-9},6\times{10}^{-9},8\times{10}^{-9}$, and the phylogenetic tree was then utilized to estimate the transmission rate $\omega$, and the simulation was repeated 100 times, and the MSE and CV of the transmission rate estimates were calculated.

In this simulation, we considered two scenarios: (i) the phylogenetic tree was constructed from all transmissions simulated by the two-population SIR model and (ii) the phylogenetic tree was constructed from a sample of transmissions simulated by the two-population SIR model. For Scenario 1, the MSE of the transmission rate estimates increases as the true value of the transmission rate grows from $2\times{10}^{-7}$ to $8\times{10}^{-7}$ (Fig. 2). The MSEs for the population size of 10,000 are very small $\left(<2\times{10}^{-14}\right)$ (Fig. 2A). Similar results can be observed for the population size of 1,000,000 (Fig. 2B), indicating that transRate can accurately estimate the transmission rate ${\omega}_{12}$ when the phylogenetic tree of all transmissions is given. The coefficient of variation (CV, i.e. the ratio of the standard deviation to the mean) of the estimates of the transmission rate ${\omega}_{12}$ for the population size of 1,000,000 (Fig. 2B) is less than those for the population size of 10,000 (Fig. 2A). This result is consistent with our theory that increasing the population size leads to a more accurate estimate of the transmission rate. For Scenario 2 where the phylogenetic tree was constructed from a sample of transmissions, the MSE of the transmission rate estimates appears to decrease as the sample size increases from 100 to 1000 (Fig. 3A), indicating that transRate can accurately estimate the transmission rate when the sample size is large. This rate of decrease varies across different values $\left(2\times{10}^{-7},4\times{10}^{-7},6\times{10}^{-7},8\times{10}^{-7}\right)$ of the transmission rate ${\omega}_{12}$. Notably, the rate of decrease appears to be constant across different values of the transmission rate $\omega$ (see Fig. 3A and B). Moreover, the MSE of the transmission rate estimates decreases at a faster rate when the sample size increases from 100 to 200, then it becomes stable after the sample size increases to 400 (Fig. 3A and b). The CV of the transmission rate estimates is less than 0.45 for the sample size $\ge 200$, suggesting that the sample size 200 is sufficient to accurately estimate the transmission rate when the phylogenetic tree of a sample of transmissions is given.

Similar results are obtained for the simulation with reduced infection and recovery rates. When the phylogenetic tree encompasses all transmissions, the MSEs of transmission rate estimates are below $3\times{10}^{-17}$ (S6a in Supplementary data). When the phylogenetic tree is derived from a sample of transmissions, the MSEs of transmission rate estimates tend to decrease as the sample size increases (S6b in Supplementary data). Moreover, the MSEs decline more rapidly as the sample size increases from 100 to 400 (S6 in Supplementary data). The CV of transmission rate estimates is less than 0.4 for sample sizes $\ge 200$ (S6 in Supplementary data), suggesting that a sample size of 200 is adequate for accurately estimating transmission rates when provided with a sample of transmissions.

#### Estimation of transmission rates from sequences

**Figure 4:**
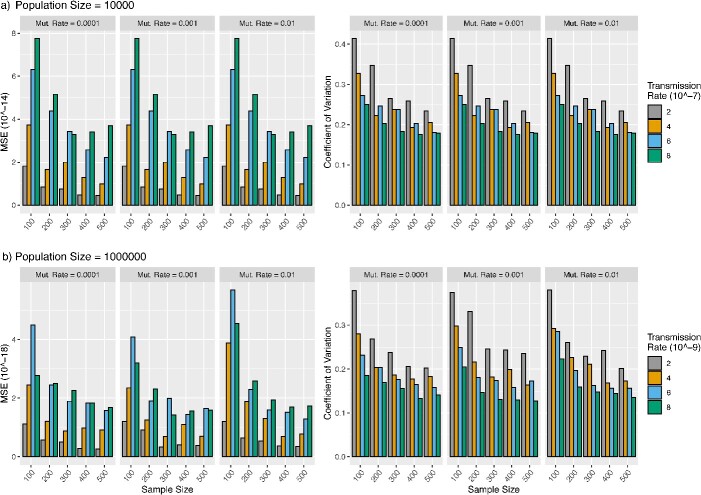
Estimation of the transmission rate from sequences; the phylogenetic tree of a sample of infected individuals (sample size = 100, 200, 300, 400, 500) was generated from the two-population SIR model; (a) for population size =  10,000, transmission events were simulated with the transmission rate $\omega =2\times{10}^{-7},4\times{10}^{-7},6\times{10}^{-7},8\times{10}^{-7}$, and (b) for population size = 1,000,000 transmission events were simulated with the transmission rate $\omega =2\times{10}^{-9},4\times{10}^{-9},6\times{10}^{-9},8\times{10}^{-9}$, and DNA sequences of 20,000 base pairs were simulated from the phylogenetic tree with the mutation rate = 0.0001, 0.001, 0.01 and then used to build the ML trees; finally, the transmission rate $\omega$ was estimated by transRate using ML trees; the simulation was repeated 100 times; the MSE and CV of the transmission rate estimates $\hat{\omega}$ were calculated.

In this simulation, we assess the accuracy of transRate in the presence of uncertainty of the estimated transmission tree. We employed the two-population SIR model to generate the transmission tree for a sample of transmissions, followed by simulating DNA sequences to reconstruct the ML trees. These ML trees were then utilized to estimate the transmission rate. The simulation results, based on a population size of 10,000 individuals, indicate that the MSE of the transmission rate estimate decreases as the sample size grows from 100 to 500 (Fig. 4). The rate of decrease appears to be positively correlated with the values of the transmission rate (Fig. 4). Moreover, the MSE of the transmission rate estimates decreases at a faster rate when the sample size grows from 100 to 200. In contrast, the MSE of the transmission rate estimates remains consistent across various values $\left(0.0001,0.001,0.01\right)$ of the mutation rate. The CV of the transmission rate estimates is less than 0.3 when the sample size increases to 200, indicating that the sample size 200 is sufficient for accurately estimating the transmission rate. The MSE and CV of transmission rate estimates for the population size 1,000,000 are less than those for the population size 10,000, which is consistent with the expectation of the multi-population SIR model that increasing the population size leads to a more accurate estimate of the transmission rate.

#### Estimation of transmission rates for five populations

**Figure 5:**
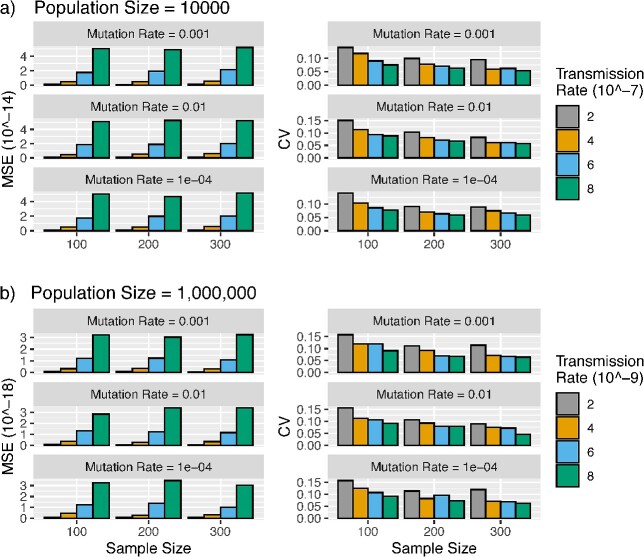
Estimation of the transmission rate for five populations; the phylogenetic tree of a sample of infected individuals (sample size = 100, 200, 300) was generated from the multi-population SIR model for five populations, and (a) for the population size = 10,000, transmission events were simulated with the transmission rate $\omega =2\times{10}^{-7},4\times{10}^{-7},6\times{10}^{-7},8\times{10}^{-7}$, and (b) for the population size = 1,000,000, transmission events were simulated with the transmission rate $\omega =2\times{10}^{-9},4\times{10}^{-9},6\times{10}^{-9},8\times{10}^{-9}$, and DNA sequences of 20,000 base pairs were simulated from the phylogenetic tree with the mutation rate = 0.0001, 0.001, 0.01 and then used to build the ML trees; finally, the transmission rate was estimated by transRate using ML trees; the simulation was repeated 100 times, and the MSE and CV of the transmission rate estimates $\hat{\omega}$ were calculated.

In this simulation, transmission events were generated from the five-population SIR model. It was assumed that all of 20 inter-population transmission rates ${\omega}_{ij}$ for $i,j=1,\dots, 5$ and $i\ne j$ were equal. For the population size of 10,000, ${\omega}_{ij}=2\times{10}^{-7},4\times{10}^{-7},6\times{10}^{-7},8\times{10}^{-7}$. For the population size of 1,000,000, ${\omega}_{ij}=2\times{10}^{-9},4\times{10}^{-9},6\times{10}^{-9},8\times{10}^{-9}$. To evaluate the performance of transRate for estimating the transmission rate, we calculated the MSE and CV of the average $\overline{\omega}$ of the estimates of 20 transmission rates. The MSE of the average estimate $\overline{\omega}$ appears to be constant as the sample size increases from 100 to 300 (Fig. 5A and B). For the population size of 10,000 and 1,000,000, the CV of the transmission rate estimates is less than 0.15 across various values $\left(0.0001,0.001,0.01\right)$ of the mutation rate and the population size ( 10,000 and 1,000,000), indicating that the sample size of 100 is sufficient for accurately estimating the transmission rate.

### Data analysis of SARS-CoV-2 genomes in the early pandemic

The transmission rate estimates for the early SARS-CoV-2 pandemic reveal transmission between Europe, Africa, Americas, Oceania, and Asia (Table 1). Population 1 is the population in which the majority of the clade is geographically centered. Population 2 is the population in which geographical outliers originate from. The analysis of the 40,028 whole genome sequences of SARS-CoV-2 in human hosts revealed the efficiency of certain protection measures enacted by public health officials. The findings suggest that, by the time many travel restrictions were in place, much transmission had already occurred between populations and with unstable availability in testing, inter-population transmission rapidly increased.

**Table 1:** Inter-population transmission rate estimates of 40,028 whole genome SARS-CoV-2 sequences

	Africa	Asia	Americas	Europe	Oceania
Africa	—				
Asia		—	2.205031e−09		6.178179e−07
Americas		1.562009e−09	—	1.01126e−08	7.398807e−08
Europe	1.123952e−07	6.297804e−10	1.414143e−09	—	1.337251e−07
Oceania			2.255848e−08	9.023393e−08	—

**Figure 6:**
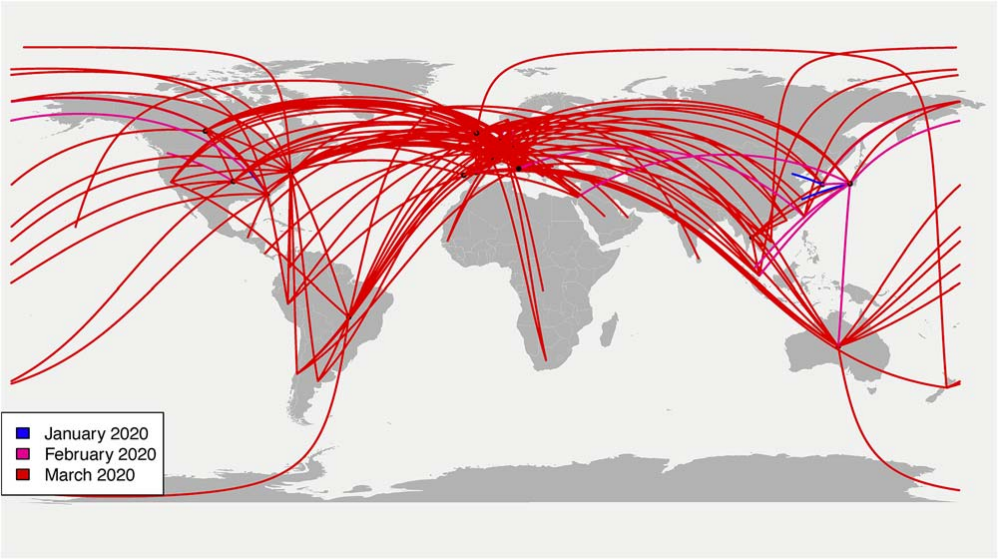
An airplane plot of transmission analysis of 40,028 whole genome sequences of SRAS-CoV-2 in human hosts between 31 December 2019 and 1 April 2020; the larger black dots on the map represent the geographical location of clades formed based on bootstrap support values and locality identity; the smaller black dots are geographical outliers in the clade and the arcs connecting the clade center to the geographical outliers represent an inferred transmission event; the color of the arcs indicates the time point in which the inferred transmission event occurred; transmission events are categorized into three time points: January 2020, February 2020, and March 2020, and the geographic coordinates were taken as the closest non-transmission taxon to the transmissions within a clade as an inference for “case 0” in a particular clade.

The airplane plot displays the inter-population transmissions of SARS-CoV-2 in human hosts between 31 December 2019 and 1 April 2020 (Fig. 6). The geographic coordinates are taken as the closest non-transmission taxon to the transmissions within a clade as an inference for “case 0” in a particular clade. The arcs indicate early transmission throughout Asia and spreading to Europe. Later transmission events are pictured from Europe the Americas. The latest transmission events shown in the airplane plot are within Oceania.

## Discussion

Estimating transmission rate is a challenging but essential task for understanding and controlling the spread of infectious diseases. This paper introduces a phylogenetic approach (transRate) to estimate inter-population transmission rates using pathogen genomes. The accuracy of transRate is influenced by the number of pathogen genomes that have been sampled from different populations. This underscores the importance of conducting higher rates of whole genome sequencing during outbreak events. However, the availability of data can present significant gaps, as not all sequences may be publicly accessible. For instance, while the dataset for SARS-CoV-2 mentioned here is extensive, there can still be biases in the data, particularly in the early stages of a pandemic [29]. The release policies for viral genomes can vary greatly between countries and change over time, as observed during the 2020 pandemic. Consequently, the sample is no longer random. Additionally, there are regions around the world where limited resources hinder the acquisition of viral genomes, as mentioned earlier. This can create limitations, especially when it comes to analyzing non-simulated data.

Upon data analysis, there is an alarming lack of data availability during the COVID-19 pandemic. A collection of all publicly available sequence from December 2019 to March 2020 was pulled in late 2020. That totaled less than 6000 samples; however, when the same query was searched in late 2021 nearly 50,000 samples resulted. This slow release of publicly available data is a hindrance in the development of tools such as transRate to assess transmission data. Another critical factor is the presence of asymptomatic cases in viral infections. Asymptomatic individuals may not receive whole genome testing, which introduces further challenges to the accuracy of the phylogenetic method.

The performance of the phylogenetic approach relies on precisely identifying populations through well-supported clades in the inferred phylogenetic tree. Low-quality sequence data can introduce considerable uncertainty in tree estimation, compromising the accuracy of transRate in transmission rate estimation. Moreover, the multi-population SIR model assumes a consistent transmission rate, whereas real-world transmission rates can vary. Hence, adopting a more advanced model that accounts for time-varying transmission rates is essential to accurately capture the evolving dynamics of transmission events.

## Conclusion

Molecular epidemiology and genetic data play a crucial role in estimating transmission rates, providing a detailed understanding of the genetic diversity and dynamics of infectious agents. The phylogenetic approach developed in this paper integrates genetic information with traditional epidemiological approaches to improve the accuracy of transmission rate estimates. Simulation and analytic results indicate that transRate can accurately estimate transmission rates from genomic data, contributing to more effective strategies for disease control and prevention. This method is well-suited for estimating transmission rates on large multi-population datasets in both epidemic and endemic states. With the increasing availability of public databases for genomic sequences, this methodology is expected to become more prevalent as a valuable policy tool.

**Key Points**

- Development of an innovative phylogenetic method to estimate transmission rates of infectious diseases.
- The phylogenetic approach is statistically consistent in estimating transmission rates under the multi-population SIR model.
- Simulation studies confirm the accuracy of the phylogenetic method in estimating transmission rates.
- The utilization of this phylogenetic approach enhances the efficacy of disease control and prevention strategies.

## Supplementary Material

Supplementary_data_bbae312

## Data Availability

The datasets analyzed for this study can be found in “The species coalescent indicates possible bat and pangolin origins of the COVID-19 pandemic” [29]. R code generated for the simulation study is available on Github at https://github.com/sagay2022/Phylogenetic-inference-of-inter-population-transmission-rates-for-infectious-diseases.

## Author Biographies

**Skylar Gay** is an undergraduate student and ThinkSwiss Scholar conducting research in the Institute of Bioinformatics at the University of Georgia.

**Gregory Ellison** is a PhD candidate in the Department of Statistics at the University of Georgia.

**Jianing Xu** is a PhD candidate in the Department of Statistics at the University of Georgia.

**Jialin Yang** is a PhD candidate in the Department of Statistics at the University of Georgia.

**Yiliang Wei** is an Associate Professor of Jiangsu Key Laboratory of Phylogenomics and Comparative Genomics in School of Life Sciences at Jiangsu Normal University.

**Shaoyuan Wu** is a Professor of Jiangsu Key Laboratory of Phylogenomics and Comparative Genomics in School of Life Sciences at Jiangsu Normal University.

**Lili Yu** is a Professor in the Department of Biostatistics at Georgia Southern University.

**Christopher Whalen** is Karen and Jim Holbrook Professor of Global Health Institute in College of Public Health at the University of Georgia.

**Jonathan Arnold** is a Professor in the Department of Genetics at the University of Georgia.

**Liang Liu** is a Professor in the Department of Statistics at the University of Georgia.
