# Supplementary material for: Phylogenetic inference of inter-population transmission rates for infectious diseases
Source: Brief Bioinform. 2024 Jun 26;25(4):bbae312. doi: 10.1093/bib/bbae312 (PMC11200198; doi:10.1093/bib/bbae312)
Supplement: Supplementary_data_bbae312 [file supplementary_data_bbae312.docx]

**Supplementary data**

## S1: Modelling transmissions within a population

A population of size $N$ consists of three components – susceptible, infectious, and recovered individuals. Let $S_{t}$ be the number of susceptible individuals at time $t$, and $I_{t}$ is the number of infectious individuals at time $t$, and $R_{t}$ is the number of recovered individuals at time $t$. It is assumed that initially there is only one infectious individual at time $0$ (i.e., $I_{0}=1$) while the number of susceptible individuals is $N-1$ at time 0 (i.e., $S_{0}=N-1$). This infectious individual $O$ at time 0 is the origin of all infections occurring in the population. The susceptible-infectious-recovered (SIR) model assumes that the dynamic flows between susceptible, infectious and recovered groups during a time interval $t\in[0,d]$ satisfy the following differential equations

$$\left\{ \begin{aligned} \frac{dS_{t}}{dt}=-\beta I_{t}S_{t} \\ \frac{dI_{t}}{dt}=\beta I_{t}S_{t}-\gamma I_{t} \\ \frac{dR_{t}}{dt}=\gamma I_{t} \end{aligned} \right. (1)$$

where $\beta$ is the infectious contact rate (i.e., the infection rate) and $\gamma$ is the recovery rate. The variables $(S_{t},I_{t},R_{t})$ are functions of time $t$, the infection rate $\beta$ and the recovery rate $\gamma$, which can be obtained by solving the differential equations in (1) with the initial condition $S_{0}=N-1$, $I_{0}=1$, and $R_{0}=0$, subject to the constraint $S_{t}+I_{t}+R_{t}=N$ for all time $t\in[0,d]$ (Figure 1).

Additionally, transmissions occur between the $S_{t}$ susceptible individuals at time $t$ and the $I_{t-1}$ infectious individuals at time $t-1$. Every newly infected individual $\mathcal{I}_{t}$ at time $t$ can be traced back to an infectious individual $\mathcal{A}_{t-1}(\mathcal{I}_{t})$ at time $t-1$. We assume that the $I_{t-1}$ infectious individuals at time $t-1$ are equally likely to be the ancestor $\mathcal{A}_{t-1}(\mathcal{I}_{t})$ of a newly infected individual $\mathcal{I}_{t}$ at time $t$, i.e., for $a=1,\ldots,I_{t-1}$

$$P\left( \mathcal{A}_{t-1}\left( \mathcal{I}_{t} \right)=a \right)=\frac{1}{I_{t-1}} (2)$$

where $a$ represents one of the $I_{t-1}$ infectious individuals at time $t-1$. All transmissions within an arbitrary time interval [0, $d^{*}$] for $d^{*}>2$ and $d^{*}\mathbb{\in N}$ form a tree-like structure, for which we call a transmission tree. The first infectious individual $O$ at time 0 is the root of the transmission tree.

**S2:** $\hat{\boldsymbol{\omega}_{\boldsymbol{12}}}\boldsymbol{=}\frac{\sum_{\boldsymbol{t}} \boldsymbol{x}_{\boldsymbol{t,2}}}{\boldsymbol{N}_{\boldsymbol{2}}\sum_{\boldsymbol{t}} \boldsymbol{I}_{\boldsymbol{t,1}}}$ **is an unbiased estimator of** $\boldsymbol{\omega}_{\boldsymbol{12}}$**.**

Proof: $E\left( \hat{\omega_{12}} \right)=E\left( \frac{\sum_{t} x_{t,2}}{N_{2}\sum_{t} I_{t,1}} \right)=\frac{E\left( \sum_{t} x_{t,2} \right)}{N_{2}\sum_{t} I_{t,1}}=\frac{\omega_{12}N_{2}\sum_{t} I_{t,1}}{N_{2}\sum_{t} I_{t,1}}=\omega_{12}$. Thus, $\hat{\omega_{12}}$ is an unbiased estimator of $\omega_{12}$.

**S3:** $\hat{\boldsymbol{\omega}_{\boldsymbol{12}}}\boldsymbol{=}\frac{\sum_{\boldsymbol{t}} \boldsymbol{x}_{\boldsymbol{t,2}}}{\boldsymbol{N}_{\boldsymbol{2}}\sum_{\boldsymbol{t}} \boldsymbol{I}_{\boldsymbol{t,1}}}$ **is statistically consistent in estimating the parameter** $\boldsymbol{\omega}_{\boldsymbol{12}}$ **as the population size** $\boldsymbol{N}_{\boldsymbol{2}}$ **goes to infinity.**

Proof: The variance of $\hat{\omega_{12}}$ is given by $Var\left( \hat{\omega_{12}} \right)=\frac{\sum_{t} Var\left( x_{t,2} \right)}{\left( N_{2}\sum_{t} I_{t,1} \right)^{2}}$ where $Var\left( x_{t,2} \right)=E\left( Var\left( x_{t,2} | y_{t,2} \right) \right)+Var\left( E\left( x_{t,2} | y_{t,2} \right) \right)=N_{2}I_{t,1}wv\left( 1-v \right)+v^{2}I_{t,1}^{2}N_{2}w\left( 1-w \right)$. Thus,

$$Var\left( \hat{\omega_{12}} \right)=\frac{I_{t,1}wv\left( 1-v \right)+v^{2}I_{t,1}^{2}w\left( 1-w \right)}{N_{2}\left( \sum_{t} I_{t,1} \right)^{2}}$$

which goes to 0 as $N_{2}$ goes to infinity. The Chebyshev's theorem indicates that $P\left( \left( \hat{\omega_{12}}-\omega_{12} \right)^{2}\geq k^{2}Var\left( \hat{\omega_{12}} \right) \right)\leq\frac{1}{k^{2}}$ for all $k>0$. Let $\epsilon=k^{2}Var\left( \hat{\omega_{12}} \right)$. Then, $P\left( \left( \hat{\omega_{12}}-\omega_{12} \right)^{2}\geq\epsilon\right)\leq\frac{Var\left( \hat{\omega_{12}} \right)}{\epsilon}$. As $N_{2}\to\infty$, we have $Var\left( \hat{\omega_{12}} \right)\to0$ and $\frac{Var\left( \hat{\omega_{12}} \right)}{\epsilon}\to0$ for all $\epsilon>0$, indicating that for all $\epsilon>0$,$\lim_{N_{2}\to\infty} P\left( \left( \hat{\omega_{12}}-\omega_{12} \right)^{2}\geq\epsilon\right)=0$. Thus, $\hat{\omega_{12}}$ is statistically consistent in estimating $\omega_{12}$.

**S4:** $\tilde{\boldsymbol{\omega}_{\boldsymbol{12}}}\boldsymbol{=}\frac{\frac{\boldsymbol{I}_{\boldsymbol{2}}}{\tilde{\boldsymbol{I}_{\boldsymbol{2}}}}\sum_{\boldsymbol{t}} {\tilde{\boldsymbol{x}}}_{\boldsymbol{t,2}}}{\boldsymbol{N}_{\boldsymbol{2}}\left( \frac{\boldsymbol{I}_{\boldsymbol{1}}}{\tilde{\boldsymbol{I}_{\boldsymbol{1}}}}\sum_{\boldsymbol{i=1}}^{\tilde{\boldsymbol{I}_{\boldsymbol{1}}}} \left( \boldsymbol{t}_{\boldsymbol{i,1}}^{\boldsymbol{R}}\boldsymbol{-}\boldsymbol{t}_{\boldsymbol{i,1}}^{\boldsymbol{I}} \right) \right)}$ **is statistically consistent in estimating the parameter** $\boldsymbol{\omega}_{\boldsymbol{12}}$ **as the population size** $\boldsymbol{N}_{\boldsymbol{2}}$ **goes to infinity.**

Let $n_{1}$ and $n_{2}$ be the sample size of $\mathcal{S}_{1}$ and $\mathcal{S}_{2}$, respectively. Additionally, $\tilde{x}_{t,i}$ denotes the number of individuals from the population $\Omega_{i}$ who travel to and get infected in the other population $\Omega_{\left| i-2 \right|+1}$ at time $t$ in the sample $\mathcal{S}_{i}$. Let $\tilde{I_{i}}$ for $i=1, 2$ be the number of the infected individuals of the population $\Omega_{i}$ in the samples $\mathcal{S}_{i}$. Note that $n_{i}=\tilde{I_{i}}+\sum_{t} \tilde{x}_{t,i}$. Let $I_{i}$ be the total number of infected individuals by the time $d$ in the populations $\Omega_{i}$. The inter-population transmission rate $\omega_{12}$ can be estimated by the samples $\mathcal{S}_{1}$ and $\mathcal{S}_{2}$ as follows

$$\tilde{\omega_{12}}=\frac{\frac{I_{2}}{\tilde{I_{2}}}\sum_{t} \tilde{x}_{t,2}}{N_{2}\left( \frac{I_{1}}{\tilde{I_{1}}}\sum_{i=1}^{\tilde{I_{1}}} \left( t_{i,1}^{R}-t_{i,1}^{I} \right) \right)} (3)$$

As the sample size $n_{2}$ approaches to the total number $I_{2}+\sum_{t} x_{t,2}$ of the infected individuals in the population $\Omega_{2}$, $\tilde{x}_{t,2}\to x_{t,2}$ and $\tilde{I_{2}}=n_{2}-\sum_{t} \tilde{x}_{t,i}\to I_{2}+\sum_{t} x_{t,2}-\sum_{t} x_{t,2}=I_{2}$. Consequently, the numerator $\frac{I_{2}}{\tilde{I}_{2}}\sum_{t} \tilde{x}_{t,2}$ in the equation (12) converges to $\frac{I_{2}}{I_{2}}\sum_{t} x_{t,2}=\sum_{t} x_{t,2}$, i.e.,

$$\frac{I_{2}}{\tilde{I_{2}}}\sum_{t} \tilde{x}_{t,2}\to\sum_{t} x_{t,2}$$

Similarly, as the sample size $n_{1}$ approaches to the total number $I_{1}+\sum_{t} x_{t,1}$ of the infected individuals in the population $\Omega_{1}$, $\tilde{x}_{t,1}\to x_{t,1}$ and $\tilde{I}_{1}\to I_{1}$. The denominator in the equation (3) converges to the denominator in $\hat{\omega_{12}}=\frac{\sum_{t} x_{t,2}}{N_{2}\sum_{i=1}^{I_{1}} \left( t_{i,1}^{R}-t_{i}^{I} \right)}$, i.e.,

$$\frac{I_{1}}{\tilde{I_{1}}}\sum_{i=1}^{\tilde{I_{1}}} \left( t_{i,1}^{R}-t_{i,1}^{I} \right)\to\sum_{i=1}^{I_{1}} \left( t_{i,1}^{R}-t_{i,1}^{I} \right)$$

Therefore, the estimate $\tilde{\omega_{12}}$ converges to $\hat{\omega_{12}}=\frac{\sum_{t} x_{t,2}}{N_{2}\sum_{i=1}^{I_{1}} \left( t_{i,1}^{R}-t_{i}^{I} \right)}$ as the sample sizes $n_{1}$and $n_{2}$ approach to the total number of the infected individuals in the populations $\Omega_{1}$ and $\Omega_{2}$, i.e.,

$$\tilde{\omega_{12}}\to\hat{\omega_{12}}$$

Similarly, we can show that $\tilde{\omega_{12}}$ (or $\tilde{\omega_{21}}$) is an asymptotically unbiased estimator of $\omega_{12}$ (or $\omega_{21}$) and is statistically consistent in estimating the parameter $\omega_{12}$ (or $\omega_{21}$) as the sample sizes $n_{1}$ and $n_{2}$ increase to infinity.

**S5: The numbers of susceptible (sky blue), infected (blue), and recovered (red) individuals at time** $\boldsymbol{t}$ **(day) were obtained by solving the differential equations for the SIR model with the infection rate** $\boldsymbol{\beta}\boldsymbol{=5\times}\mathbf{10}^{\mathbf{-6}}$ **and the recovery rate** $\boldsymbol{\gamma}\mathbf{=0.005}$**.**

**S6: Simulation for a population of 10,000 with reduced infection and recovery rates** $\beta\boldsymbol{=5\times}\mathbf{10}^{\mathbf{-6}}$ **and** $\boldsymbol{\gamma}\mathbf{=0.005}$**. a) Estimation of the transmission rate from the phylogenetic tree of all infected individuals. Transmission events were simulated with the transmission rate** $\boldsymbol{\omega}\boldsymbol{=2\times}\mathbf{10}^{\mathbf{-8}}\boldsymbol{, 4\times}\mathbf{10}^{\mathbf{-8}}\boldsymbol{, 6\times}\mathbf{10}^{\mathbf{-8}}\boldsymbol{, 8\times}\mathbf{10}^{\mathbf{-8}}$**. The simulation was repeated 100 times. The Mean Squared Error (MSE) and Coefficient Variation (CV) of the transmission rate estimates were calculated. b) Estimation of the transmission rate from the phylogenetic tree of a sample of infected individuals. The phylogenetic tree of a sample of infected individuals (sample size = 100, 200, 400, 600, 800, 10000) was generated from the two-population SIR model.**

**S7: Clades assembled during analysis of 40,028 whole genome SARS-CoV-2 sequences.**

| Geographic Clade Majority | Number of Clades | Total Sequences in Clades |
| --- | --- | --- |
| United States of America | 8 | 2,701 |
| Switzerland | 1 | 334 |
| United Kingdom | 4 | 246 |
| Netherlands | 2 | 237 |
| Italy | 3 | 218 |
| Iceland | 2 | 203 |
| Canada | 2 | 191 |
| Japan | 3 | 189 |
| Thailand | 1 | 130 |
| Australia | 1 | 111 |
| Spain | 1 | 103 |
| Denmark | 1 | 79 |
| Brazil | 1 | 66 |
| Sweden | 1 | 64 |
| South Korea | 1 | 61 |
| Germany | 1 | 55 |
| Portugal | 1 | 54 |
| France | 1 | 52 |
| Total: | 35 | 5,094 |

**S8: Input data for real-data analysis.**

| Population 1 | Population 2 | I_2_ | n_2_ | N_2_ | sig | I_1_ |
| --- | --- | --- | --- | --- | --- | --- |
| Africa | Asia | 155073 | 0 | 4664324075 | 0 | 3786 |
|  | Americas | 163014 | 0 | 1025792795 | 0 | 3786 |
|  | Europe | 423946 | 0 | 746189645 | 0 | 3786 |
|  | Oceania | 4359 | 0 | 43933425 | 0 | 3786 |
| Asia | Africa | 3786 | 0 | 1360677231 | 380 | 155073 |
|  | Americas | 163014 | 12 | 1025792795 | 380 | 155073 |
|  | Europe | 423946 | 0 | 746189645 | 380 | 155073 |
|  | Oceania | 4359 | 1 | 43933425 | 380 | 155073 |
| Americas | Africa | 3786 | 0 | 1360677231 | 2958 | 163014 |
|  | Asia | 155073 | 29 | 4664324075 | 2958 | 163014 |
|  | Europe | 423946 | 28 | 746189645 | 2958 | 163014 |
|  | Oceania | 4359 | 65 | 43933425 | 2958 | 163014 |
| Europe | Africa | 3786 | 8 | 1360677231 | 1645 | 423946 |
|  | Asia | 155073 | 40 | 4664324075 | 1645 | 423946 |
|  | Americas | 163014 | 81 | 1025792795 | 1645 | 423946 |
|  | Oceania | 4359 | 20 | 43933425 | 1645 | 423946 |
| Oceania | Africa | 3786 | 0 | 1360677231 | 111 | 4359 |
|  | Asia | 155073 | 0 | 4664324075 | 111 | 4359 |
|  | Americas | 163014 | 8 | 1025792795 | 111 | 4359 |
|  | Europe | 423946 | 2 | 43933425 | 111 | 4359 |
